# Effect of *Mobola* Plum (*Parinari curatellifolia*) Flour Incorporation on Physicochemical, Nutritional, and Antioxidant Properties of Cornstarch‐Based Composite Flours

**DOI:** 10.1002/fsn3.72259

**Published:** 2026-08-11

**Authors:** Shonisani E. Ramashia, Winnie Ndou, Oluwaseun P. Bamidele

**Affiliations:** ^1^ Department of Food Science and Technology University of Venda Thohoyandou South Africa

**Keywords:** antioxidant, color, fruit flour, mineral, proximate composition

## Abstract

The addition of underused indigenous fruit flours in starch‐centred systems has the potential to make staple foods more nutritionally and functionally diverse. This study determines the effects of adding *M*obola plum (*Parinari curatellifolia*) flour on the physicochemical, nutritional, and antioxidant properties of cornstarch. The control was 100% cornstarch, and the substitution ranged from 10% to 50% (w/w) *M*obola plum flour. The addition of *Mobola* plum flour resulted in increases in pH (5.51–6.03) and total soluble solids (0.27–3.12 °Brix), while total titratable acidity remained relatively stable. The increase in the percentage of *M*obola plum flour significantly improved the functional attributes, total phenolic content, antioxidant activities and color attributes of the composite flour. Pasting attributes of peak, final and setback viscosity were significantly reduced, and pasting temperature was significantly increased. The study results show that *M*obola plum flour enhances the functional and antioxidant properties of composite flours at 10%–30% substitution levels. It is therefore possible to use *M*obola plum flour in the formulation of gluten‐free, functional, value‐added food products.

## Graphical abstract

*Mobola* plum fruit flour addition to corn starch increases the physicochemical properties of the corn starch. It reduced the protein content of the corn starch after addition. *Mobola* plum fruit flour addition to corn starch increased the TPC and antioxidant activities of corn starch. The higher the percentage of *Mobola* plum fruit flour to corn starch, the better the TPC and the antioxidant activities of corn starch.

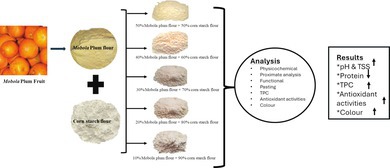

## 1 Introduction

The use of composite flours based on starches has been investigated in food science and technology for their ability to improve the nutritional value, functionality, and sustainability of food products while circumventing problems associated with mono‐cereal ingredients (Yuan et al. 2025). Starch from corn ( *Zea mays* ) is one of the most commercially available and preferred starches due to its neutral taste and its cheap, effective thickening and gelling properties (Khatefov et al. 2023). The nutritional value of maize starch is inadequate because the protein, dietary fiber, and mineral content, as well as the bioactive (phenolic and flavonoid) compounds, are deficient. Therefore, the use of maize starch in the formulation of health‐promoting foods is limited (Meena et al. 2022). To solve this problem, researchers have attempted to strengthen the physiochemical, nutritional, and functional properties of composite flours by incorporating some nutrient‐rich and underutilized native plant (fruit) flours into the starch matrices.

The *Mobola* Plum (*Parinari curatellifolia*) is a wild native tree grown in Eastern and Southern Africa (Kaseke et al. 2025), and as a result, is an important tree species for the possible agroforestry enrichment of southern Africa. The wild *Mobola* Plum fruits are highly appreciated by rural communities as both a source of food and for its cultural significance (filled with sweet–sour pulp), and the fruits have a nutritional profile that includes carbohydrates, dietary fiber, and are especially high in vitamin C, as well as important minerals such as calcium, magnesium, and potassium, and the fruits contain some polyphenolic compounds that have antioxidant activity (Sibiya 2019; Ndhlovu et al. 2024). Furthermore, the dry pulp of the fruit can be processed into ‘*Mobola* plum flour’, a raw material with the potential to increase food security and economic value in the areas where the tree grows (as it is largely unutilized). There is a wealth of nutritional research that has analyzed the *Mobola* Plum and its flour; preliminary results have shown high phenolic compounds and scavenging activities in the flour, which are due to the presence of certain flavonoids, tannins, and anthocyanins, and suggest that the flour has the potential to exhibit anti‐inflammatory and anti‐diabetic activities (Kaseke et al. 2025).

The addition of *Mobola* plum flour to maize starch‐based composites will alter pasting behavior, water and oil absorption, gel texture and color, while increasing the protein, fiber, mineral and antioxidant content. These changes are important for the development of gluten‐free products, functional snacks and weaning foods aimed at people who are more likely to suffer from micronutrient deficiencies and oxidative stress‐related disorders in sub‐Saharan Africa. In addition, the combination of indigenous flours and maize starch aligns with global efforts to promote the sustainable use of biodiversity‐rich crops and increased dietary diversification (Kaur et al. 2025).

There is still a lack of understanding of the functional and nutritional synergies of *Mobola* plum flour and maize starch in composite systems, as well as their impact on optimal blending ratios and product outcomes during processing (e.g., extrusion and baking). Therefore, the objective of this study is to provide scientific evidence on the effects of *Mobola* plum (*Parinari curatellifolia*) flour on the physicochemical properties, nutritional profile, and antioxidant activity of maize starch‐based composite flours, to support the creation of innovative, nutrient‐dense food products from untapped African resources.

## 2 Materials and Methods

The cornstarch was purchased from Ingrain, South Africa. Ripe *Mobola* Plum (*Parinari curatellifolia*) was harvested from the trees at the University of Venda, behind the Department of Food Science and Technology building. All the chemicals used for this study were purchased from Merck, Centurion, South Africa, and were of analytical grade.

### 2.1 *Mobola* Plum (*P. curatellifolia*) Flour Preparation

*Mobola* Plum flour was prepared according to the method described by Adeyanju and Bamidele (2022) with slight modifications. The fruits (ripe *Mobola* plum) were cleaned using running tap water and thereafter weighed. The fresh ripe *Mobola* plum fruits were peeled manually, and then the pulp was separated from the hard kernel using a stainless‐steel knife. The *Mobola* plum fruit pulp was spread onto oven trays and dried in a hot‐air oven at 50°C for 24 h. The dried pulp was crushed and milled into flour using a Retsch ZM 200 mill (Haan, Germany). It was then sieved using a 500 μm sieve. After sieving, the flour was packaged in low‐density, properly labeled polyethylene bags and stored at 4°C until further analyses.

### 2.2 Sample Formulation

**FIGURE 1:**
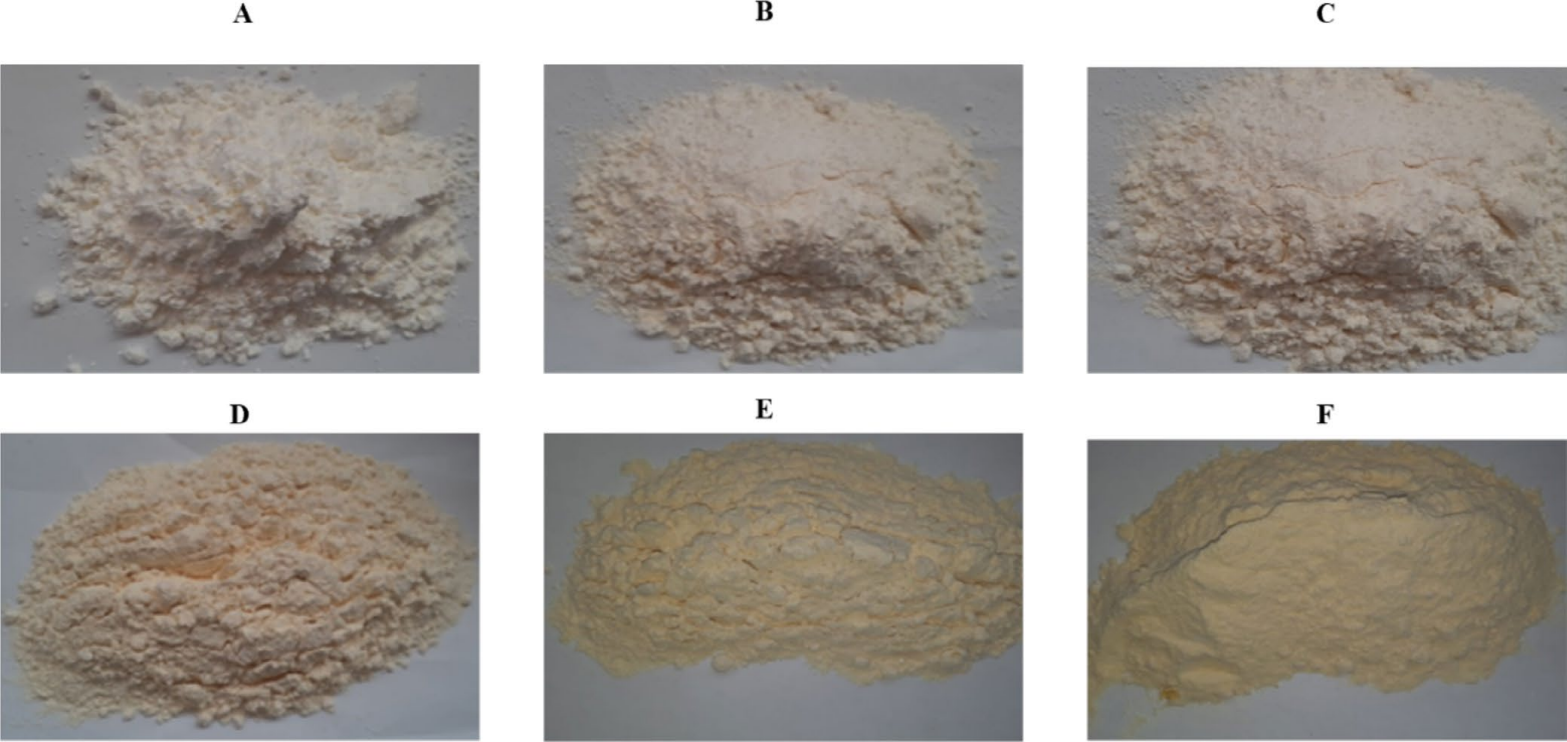
Composite flour made from a blend of cornstarch and *Mobola plum* (*Parinari curatellifolia*) flour. (A) 100% corn flour, (B) cornstarch (90%): *Mobola* plum flour (10%) (CPF1), (C) cornstarch (80%): *Mobola* plum flour (20%) (CPF2), (D) cornstarch (70%): *Mobola* plum flour (30%) (CPF3), (E) cornstarch (60%): *Mobola* plum flour (40%) (CPF4), and (F) cornstarch (50%): *Mobola* plum flour (50%) (CPF5).

The cornstarch and *Mobola* Plum flour blends were prepared at varying ratios to yield five blends. The ratios are cornstarch (90%): *Mobola* Plum flour (10%) (CPF1), cornstarch (80%): *Mobola* Plum flour (20%) (CPF2), cornstarch (70%): *Mobola* Plum flour (30%) (CPF3), cornstarch (60%): *Mobola* Plum flour (40%) (CPF4), and cornstarch (50%): *Mobola* Plum flour (50%) (CPF5). A 100% cornstarch flour served as a control. The composite flour samples were sealed in polyethylene bags and stored at 4°C for further use (Figure 1).

### 2.3 Physico‐Chemical Properties of the Composite Flour

#### 2.3.1 Determination of pH

The pH of the flour blends was determined at room temperature using a pre‐calibrated Crison digital pH Meter (Crison Instrument, South Africa). Three‐point calibration was performed using pH 10.0, 7.0, and 4.0 buffers prior to the analysis. Ten grams of the sample was immersed in 100 mL of distilled water and allowed to stand for 5 min. The pH electrode was completely submerged in the mixture, and readings were taken (Mafukata et al. 2024).

#### 2.3.2 Total Titratable Acidity (TTA)

For total titratable acidity, 100 mL of distilled water was added to 10 g of the cornstarch‐*Mobola* Plum flour blends samples, and they were kept at room temperature (25°C ± 1°C) for 30 min. The supernatant was filtered, and 10 mL was titrated with 0.1 N NaOH using phenolphthalein as the endpoint indicator (Bamidele et al. 2025).

(1) $$\begin{array}{c} \text{Titratable acidity}\;\left(\%\text{malic acid}\right)\\ \;=\frac{\text{Volume of titre}\times 0.1\;\text{NaOH}\times 0.067}{\text{Volume of Sample}}\times 100 \end{array}$$

#### 2.3.3 Total Soluble Solids (TSS)

The composite flour samples were mixed with distilled water and heated to 100°C for 1 h. Then, a drop of the powder supernatant was placed on the prism of a pre‐calibrated Leica Auto Abbe refractometer, Model 10,504 (Leica Inc., Buffalo, NY, USA), and readings were recorded in triplicate (Bamidele et al. 2025).

### 2.4 Proximate Composition

The American Association of Cereal Chemists (AACC 2000) International techniques were used to determine the proximate analyses of the composite flours; the AOAC (2005) method was used to determine the crude fiber content. For moisture content, AACC method 44‐15A was employed; AACC method 30–25 for ash content, AACC method 08‐01; and for crude protein, AACC method 46–30. The carbohydrate was calculated as the difference of:

$$100–\left(\%\text{moisture}+\%\mathrm{ash}+\text{crude fiber}+\%\text{crude}\;\mathrm{fat}+\%\text{crude protein}\right).$$

### 2.5 Functional Properties of the Composite Flour

The composite flour's functional properties, including water absorption capacity (WAC), oil absorption capacity (OAC), swelling power (SP), bulk density (BD), and water solubility index (WSI), were assessed using modified methods from Adeyanju and Bamidele (2022). Two grams of the composite flour sample were added to 30 mL of water to determine WAC. After 10 min of venting, the sample was left to rest for a further 10 min. The sample was centrifuged at 4100 *g* for 15 min. The mass of the residual (*W*_2_) was determined by weighing the tube after decanting the supernatant. Grams of water absorbed per gram of flour were used to compute WAC.

(2) $$\mathrm{WAC}=\frac{W_{2}-W_{1}}{\text{Weight of the sample}}\times 100$$

A 50 mL centrifuge tube containing 2 g of flour was used to measure the composite flour's water solubility index, swelling capacity, and oil absorption capacity (OAC). After adding 30 mL of pure water, the sample was well stirred. After mixing, the sample was heated to 90°C for 15 min in a water bath, stirred occasionally to prevent clumping. After centrifuging the paste for 10 min at 4100× *g* in an Eppendorf centrifuge (5702R, Germany), the supernatant was carefully decanted. The proportion of dry particles recovered from the supernatant after an overnight oven‐drying process at 100 degrees Celsius was used to calculate the water solubility index.

(3) $$\text{Oil absorption capacity}\;\left(g/\mathrm{mL}\right)=\mathrm{OAC}=\frac{W_{2}-W_{1}}{W_{1}}\;X\;100$$

where *W*_2_ is the weight of the oil sample, and *W*_1_ is the initial weight of the dry sample.

(4) $$\text{Solubility index}\;\left(\%\right)=\frac{\text{Weight of}\;\mathrm{dry}\;\text{solid after drying}}{\text{Weight of the sample}}\times 100$$

(5) $$\text{Swelling power}\;\left(g/g\right)=\frac{\text{weight of}\;\mathrm{wet}\;\text{mass of sediment}}{\text{weight of}\;\mathrm{dry}\;\text{matter in}\;\mathrm{gel}}$$

A 50 mL graduated cylinder containing 10 g of flour was used to measure bulk density. Until the sample volume stabilized, the sample was tapped ten times from a height of five centimeters on the bench top. After measuring the sample weight, the bulk density was calculated from the weight and the tapped volume.
(6)

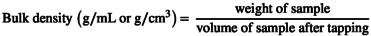




### 2.6 Pasting Properties of the Composite Flour

Evaluation of the pasting properties of composite flour samples was conducted using, with few modifications, the method described by Adeyanju and Bamidele (2022). Utilizing a Rapid Visco Analyzer (TECMASTER, Perten Instruments, Hägersten, Sweden), the pasting characteristics of the flour samples were assessed. A 30‐mL aluminium canister was filled with around 3 g of the sample, and 25 mL of pure water was added. The paddle connection was attached after the paddle was installed. The Rapid Visco Analyzer (RVA) was filled with the paddle and sample container. The instrument's motor tower was pressed to start the measurement sequence. Using the following temperature time parameters, a 14‐min profile was produced: 1 min at 50°C with the paddle driving at 960 rpm for the first 10 s and 160 rpm for the remaining 1 min hold; the temperature was then ramped to 91°C, over 4 min and held there for 3 min; it was then cooled to 50°C, over 4 min and held there for 2 min.

### 2.7 Extraction of Free Phenolic Compounds and Determination of Total Phenolic Content of the Composite Flour

Each sample was prepared in triplicate to extract free phenolic compounds from the composite flour samples. An orbital shaker (Stuart Cole‐Parmer Ltd., India) was used to shake 30 mL beakers containing 1 g of each sample for 2 h at 200 rpm (rpm). After adding 10 mL of 1% HCl‐methanol, the beakers were foiled. After centrifuging the samples for 10 min at 3500 *g*, the supernatant was poured into 30‐mL tubes. After a half‐hour soak, the residue was also centrifuged. The supernatant was then added and kept at −20°C.

The same process was followed for 70% aqueous acetone. The total phenolic content was assessed using the method of Adeyanju and Duodu (2023) with modifications. In triplicate, 0.1 mL of the 1% HCl‐methanol extract, blank (solvent), and gallic acid standard at varying concentrations (0.1–0.5 mg/mL) were added to 15 mL test tubes containing 7 mL of deionized water. 0.5 mL of Folin–Ciocalteu reagent was added to the mixture, then the incubated mixture was subjected to the absorbance measurement at 765 nm via UV spectrophotometer (Biochrom Libra PCB 1500, Biochrom Ltd., UK), after the sequential addition of 1.5 mL of 20% (w/v) sodium carbonate. The results were presented in gallic acid equivalents (GAE)/g sample.

### 2.8 Antioxidant Assays

The modified method of Bamidele et al. (2025) was used to determine the 2,2′‐azino‐bis (3‐ethylbenzothiazoline‐6‐sulfonic acid) (ABTS) radical scavenging activity of the composite flour samples. For this assay, ABTS (8 mg) was combined with deionized water (1 mL) to prepare solution A (8 mM). Then, potassium persulphate (1.32 mg) was mixed with deionized water (1 mL) to form solution B (3 mM). Equal volumes (1 mL each) of solutions A and B were mixed and left in the dark at room temperature for 12–16 h to form ABTS•^+^ radical cation. In order to achieve an absorbance of 0.7 at 734 nm for the working ABTS solution, 2 mL of the radical solution was diluted with 58 mL of phosphate‐buffered saline (PBS, pH 7.4). Next, 3 mL of the working ABTS solution was mixed with 0.2 mL of the HCl‐methanol extract, and the mixture was incubated at room temperature in the dark for 30 min. A UV spectrophotometer (Biochrom Ltd., UK) was used to measure the absorbance at 734 nm. Utilizing Trolox standards (0–1000 μM), the antioxidant capacity was calculated and reported as micromoles of Trolox equivalents per gram of sample (μmol TE/g).

The 2,2‐diphenyl‐1‐picrylhydrazyl (DPPH) radical‐scavenging method used was adapted from Bamidele and Fasogbon (2017). A stock solution of 0.609 mM DPPH was prepared by dissolving 24 mg DPPH in 100 mL of methanol. Ten milliliters of the stock solution were diluted with 50 mL of methanol to create a working solution, and the absorbance was then adjusted to approximately 1.1 at 515 nm. For this process, 3 mL of the DPPH working solution and 0.2 mL of the 70% (v/v) methanol extract were mixed. Following mixing and 30 min in the dark, the sample's absorbance was measured at 515 nm using a UV spectrophotometer (Biochrom Ltd., UK) and computed using the Trolox standard curve (0–1000 μM) and expressed as micromoles of Trolox equivalents per gram of sample (μmol TE/g).

The Ferric Reducing Antioxidant Power (FRAP) was also adapted from Adebayo et al. (2026) with slight modifications. This method is based on the color change from colorless to blue, due to the reduction of ferric (Fe^3+^) to ferrous (Fe^2+^) in the tripyridyltriazine (TPTZ) complex, resulting from electron donation by the antioxidants. For this assay, each extract sample (100 μL) was combined with 200 μL of deionized water and 2 mL of FRAP reagent, then incubated for 30 min at 37°C. The absorbance was measured at 595 nm and compared to a standard curve of Trolox, with antioxidant activity expressed as micromoles of Trolox equivalents per gram of sample (μmol TE/g).

### 2.9 Color Attributes of Flour and Custard

Following the color analysis procedures of Mafukata et al. (2024), color analysis was performed, and color quality was measured using a ColourFlex colourimeter and a HunterLab spectrophotometer (Hunter Associates Laboratory Inc., Reston, VA). Three of the samples' color attributes were measured: L* (lightness/darkness), a* (redness/greenness), and b* (yellowness/blueness). The colourimeter was calibrated with a standard white plate (L* = 93.71, a* = −0.84, and b* = 1.83) and a black plate before use (Thilagavathi et al. 2015). According to the whiteness index (WI) and yellowness index (YI). Chromatic color difference (Δ*E*) and whiteness were measured using the methods of Anyasi et al. (2017).

(7) $$\text{Chroma}=\surd {\left(a^{*}\right)}^{2}+{\left(b^{*}\right)}^{2}$$

(8) $$\mathrm{Hue}=\tan^{-1}\;\left(b/a\right)$$

(9) $$\mathrm{WI}=100-{\left[{\left(100-L^{*}\right)}^{2}+{\left(a^{*}\right)}^{2}+{\left(b^{*}\right)}^{2}\right]}^{0.5}$$

(10) $$\mathrm{YI}=\frac{142.86b}{L}$$

(11) $$\text{∆}E=\surd {\left(\text{∆}L\right)}^{2}+{\left(\text{∆}a\right)}^{2}+{\left(\text{∆}b\right)}^{2}$$

### 2.10 Statistical Analysis

Every analysis was carried out three times. The Statistical Package for the Social Sciences (IBM SPSS; version 22, Armonk, USA) was used to analyze the data using the ANOVA, General Linear Model, and Univariate methods. The LSD post hoc test was used to find significant differences between means at a 95% confidence level (*p* < 0.05).

## 3 Results and Discussions

### 3.1 Physicochemical Properties of Composite Flour Made From Cornstarch‐*Mobola* Plum (*Parinari curatellifolia*) Flour

Table 1 highlights the physicochemical characteristics of modified cornstarch‐*Mobola* plum composite flours. The data demonstrate a trend: the *Mobola* plum flour proportion increased from the control to CPF5. The control (100% cornstarch) had the lowest pH (5.51), while CPF5 had the highest pH (6.03). The increase in pH indicates a compositional shift in the predominant source of acidity, correlating with the increased quantity of *Mobola* plum flour. The *Mobola* plum flour may be composed of materials with a pH ≥ 7 or higher that may neutralize the source of acidity of the cornstarch (Ramashia et al. 2021). Although the pH values were significantly different, all values still fell within the narrow range of mildly acidic, which could be beneficial for flour stability and may also synergise with enzymatic activities to improve the flour's shelf life during storage.

**TABLE 1:** Physicochemical properties of composite flour made from cornstarch‐*Mobola* plum flour.

Parameter	pH	TSS (°Brix)	TTA (%)
Control	5.51 ± 0.7^a^	0.27 ± 0.1^a^	0.33 ± 0.1^a^
CPF1	5.71 ± 0.5^b^	0.81 ± 0.1^b^	0.47 ± 0.2^a^
CPF2	5.83 ± 0.2^c^	1.24 ± 0.1^c^	0.50 ± 0.0^a^
CPF3	5.90 ± 0.7^d^	1.77 ± 0.3^d^	0.46 ± 0.1^a^
CPF4	5.96 ± 0.6^e^	2.62 ± 0.4^e^	0.56 ± 0.1^a^
CPF5	6.03 ± 0.5^f^	3.12 ± 0.5^f^	0.34 ± 0.1^a^

*Note:* The values are mean ± SD of triplicate determinations. Values followed by the same superscript(s) within the column differ significantly (*p* ≤ 0.05). CPF1 (90:10), CPF2 (80:20), CPF3 (70:30), CPF4 (60:40) and CPF5 (50:50) Cornstarch (Control): *Parinari curatellifolia* (PC). TSS is Total Soluble Solids, TTA is Total Titratable Acidity.

The most likely reason for the observed TSS increase as *Mobola* plum flour inclusion increased, which was 0.27 °Brix for the control and 3.12 °Brix for the CPF5, is the contribution of the soluble sugars and other soluble solids from *Mobol*a plum flour, which is high in simple sugars (Kaseke et al. 2025). The substantial differences across all the compositions demonstrate how sensitive TSS is to the extent of substitution of the dependent variable (fruit flour). TSS is likely to favorably impact some functional properties, such as taste and color changes during processing (Zhang et al. 2022), as well as the overall quality of products derived from these composite flours.

Conversely, TTA values spanned from 0.33% to 0.56%, and did not differ statistically (*p* > 0.05) among the samples. This shows that the titratable acidity of the composite flours is consistent and changes little across a range of pH values. This shows that the organic acid content from *Mobola* plum flour was low or well‐buffered within the composite (Oladunjoye and Apalara 2025). This indicates that TTA is stable, making it good, as it shows little change will occur in any acid‐catalyzed reactions during processing and storage.

### 3.2 Proximate Composition of the Composite Flour

The changes in proximate composition from increasing levels of incorporation of *Mobola* plum flour into cornstarch showed in the moisture content, ash, crude protein, and crude fat, while carbohydrate (CHO) percentage did not show a statistical difference (Table 2). The moisture content for Control was 11.55%, and it progressively decreased to 4.89% for CPF5. All CPF levels were significantly lower than control (*p* < 0.05). The decline in moisture levels was due to the lower inherent moisture of *Mobola* plum flour (Ramashia et al. 2021) than cornstarch, which has a higher moisture content (Wang, Dong, et al. 2022). Higher CPF blends have lower moisture, which may improve shelf stability by reducing water activity and, in turn, less microbial growth and enzymatic reactions (Awol et al. 2023). A moisture content of CPF5 below 6% may also increase flour brittleness and reduce hydration capacity during dough formation, thereby affecting the texture of the end product (Larrosa and Otero 2021).

**TABLE 2:** Proximate composition of composite flour made from cornstarch‐*Mobola* plum flour.

Parameter	Control	CPF1	CPF2	CPF3	CPF4	CPF5
Moisture (%)	11.55 ± 0.5^d^	9.33 ± 0.8^c^	9.22 ± 0.6^c^	8.67 ± 0.5^c^	6.55 ± 0.4^b^	4.89 ± 0.7^a^
Ash (%)	1.22 ± 0.2^a^	1.33 ± 0.1^a^	2.04 ± 0.3^b^	2.15 ± 0.2^b^	2.80 ± 0.1^c^	3.23 ± 0.3^d^
Protein (%)	3.17 ± 0.2^f^	2.41 ± 0.2^c^	2.22 ± 0.3^d^	1.78 ± 0.1^c^	1.62 ± 0.1^b^	1.42 ± 0.3^a^
Fat (%)	1.71 ± 0.2^d^	1.61 ± 0.1^d^	1.53 ± 0.1^bc^	1.42 ± 0.1^b^	1.18 ± 0.1^a^	1.07 ± 0.1^a^
CHO (%)	88.93 ± 0.9^a^	88.09 ± 0.6^a^	85.54 ± 0.6^a^	85.43 ± 0.6^a^	85.06 ± 0.9^a^	86.38 ± 0.7^a^

*Note:* The values are mean ± SD of triplicate determinations. Values followed by the same superscript(s) within the column differ significantly (*p* ≤ 0.05). CPF1 (90:10), CPF2 (80:20), CPF3 (70:30), CPF4 (60:40) and CPF5 (50:50) Cornstarch (Control): *Mobola* plum (*Parinari curatellifolia*) (PC). CHO refers to carbohydrates.

The ash content of the blends rose from 1.22% ± 0.2% (control) to 3.23% ± 0.3% (CPF5), demonstrating the rich mineral content of *Mobola* plum flour (Chatepa et al. 2018). The fruit pulp is reported to be rich in potassium, calcium, magnesium, and other trace elements (Czech et al. 2020), and, with increasing levels of incorporation, raises ash content. Elevated levels of ash reflect increased mineral content; therefore, the mineral richness of CPF‐enriched flours is beneficial for combating micronutrient deficiencies in cereal‐based diets prevalent in sub‐Saharan Africa (Aloka et al. 2025).

The crude protein decreased from 3.17% ± 0.2% (control) to 1.42% ± 0.3% (CPF5), with all CPF levels significantly lower than control (*p* < 0.05). Cornstarch is low in crude protein (Jesulagba et al. 2024), while *Mobola* plum flour has lower crude protein (Kaseke et al. 2025) compared to legumes or cereals. The observed decline is therefore expected and reflects dilution of the minor protein fraction in cornstarch by *Mobola* plum flour. This reduction may limit the use of high‐CPF blends in protein‐fortified applications unless combined with protein‐rich ingredients.

The crude fat also declined from 1.71% ± 0.2% (control) to 1.07% ± 0.1% (CPF5), with significant reductions at higher CPF levels (*p* < 0.05). Cornstarch contains trace lipids, while *Mobola* plum pulp is low in fat (1%–2%), leading to progressive dilution. The low‐fat content across all blends (< 2%) is advantageous for formulating low‐fat, health‐oriented products (Tufeanu and Tița 2016). The carbohydrate content (calculated by difference) ranged from 85.06% to 88.93%, with no significant differences (*p* > 0.05). The high‐carbohydrate fraction reflects the dominance of cornstarch and sugars from the *Mobola* plum, making these composites suitable for energy‐dense foods.

### 3.3 Functional Properties of the Composite Flour

The functional properties of composite flours produced from cornstarch and *Mobola* plum flour are presented in Table 3. These properties are critical indicators of the performance of composite flours during processing and their suitability for specific food applications.

**TABLE 3:** Functional properties of composite flour made from cornstarch‐*Mobola* plum flour.

Sample	BD (g/cm^3^)	WAC (g/mL)	OAC (g/mL)	SP (g/g)
Control	0.57 ± 0.0^a^	2.02 ± 0.5^a^	2.55 ± 0.2^a^	2.39 ± 0.2^a^
CPF1	1.34 ± 0.1^b^	2.52 ± 0.3^b^	2.58 ± 0.6^a^	3.94 ± 0.5^b^
CPF2	1.38 ± 0.1^b^	2.53 ± 0.6^b^	2.63 ± 0.5^a^	4.43 ± 0.6^c^
CPF3	1.59 ± 0.1^c^	2.74 ± 0.2^b^	2.70 ± 0.4^a^	5.78 ± 0.7^d^
CPF4	1.68 ± 0.1^d^	2.79 ± 0.2^b^	3.05 ± 0.6^b^	4.44 ± 0.6^c^
CPF5	1.79 ± 0.1^d^	2.80 ± 0.2^b^	3.10 ± 0.5^b^	5.49 ± 0.7^c^

*Note:* The values are mean ± SD of triplicate determinations. Values followed by the same superscript(s) within the column differ significantly (*p* ≤ 0.05). CPF1 (90:10), CPF2 (80:20), CPF3 (70:30), CPF4 (60:40) and CPF5 (50:50) Cornstarch (Control): *Parinari curatellifolia* (PC). BD is bulk density, WAC is water absorption capacity, OAC is oil absorption capacity, and SP is swelling power.

There was a notable increase in bulk density with greater incorporation of *Mobola* plum flour, with values of 0.57 and 1.79 g/cm^3^ in the control and CPF5, respectively. The bulk density of the control is low due to cornstarch's lightweight, more porous construction, and the processed composite flours are observed to have higher bulk densities due to this construction. *Mobola* plum flour has a denser particle structure and a higher fiber content. Increased bulk density has a positive impact on the efficiency of packaging, transport, and reconstitution of the formulated food, especially in instant and complementary products, where the food's energy density is of high importance (Ren et al. 2024). There was a significant increase in water absorption capacity from 2.02 g/mL in the control to 2.80 g/mL in CPF5. This increase indicates that the composite flours have greater water‐binding capacity, particularly with a higher *Mobola* plum flour content. The increased water absorption capacity (WAC) is most likely due to the hydrophilic constituents in *Mobola* plum flour, such as fiber, polysaccharides, and proteins, which are water‐binding (Olubi et al. 2024). Better water absorption is advantageous in food systems such as doughs, batters and soups, where the water retention provides a desirable texture and improved yield (Awuchi et al. 2019).

Oil absorption capacity (OAC) increased with increasing substitution of *Mobola* plum flour, though this only became statistically evident with higher substitutions (CPF4 and CPF5). OAC in CPF5 reached 3.10 g/mL compared to 2.55 g/mL in the control. The increased oil absorption may be due to greater exposure of nonpolar protein side chains and to composite flour matrix structures that can physically entrap oil (Li et al. 2025). High OAC is advantageous for retaining flavors and mouthfeel in baked products, meat extenders, and snack formulations (Chen et al. 2023). The inclusion of *Mobola* plum flour also led to significant increases in swelling power (SP), with the control at 2.39 g/g and CPF3 at 5.78 g/g, showing only slight increases at higher substitution levels. The overall increase in SP demonstrates improved hydration and expansion of starch granules in the composite flours. The initial increase could be attributed to the synergistic combination of cornstarch and non‐starch polysaccharides from *Mobola* plum flour, which may aid in water absorption and granule swelling (He et al. 2022). The modest decrease at higher inclusion levels may reflect structural limitations imposed by increased fiber content, which may inhibit starch granule swelling (Wang, Ral, et al. 2022).

### 3.4 Pasting Properties of Composite Flour From Cornstarch and *Mobola* Plum Flour at Different Ratios

Table 4 shows the pasting properties of the composite flours made with cornstarch (CS) and *Mobola* plum flour at varying substitution levels (CPF1–CPF5). Peak viscosity (PV) shows the water‐binding ability and swelling potential of starch granules during heating. For control, the PV value was 3820.01 RVU and significantly dropped to 1650.09 RVU for CPF5. This downward trend indicates that replacing cornstarch with *Mobola* plum flour reduces the starch quantity and/or introduces non‐starch constituents, such as dietary fiber, lipids, and phenolic compounds, which inhibit granule swelling. Similar peak viscosity reductions have been observed in composite flours (Giri et al. 2022; Alija et al. 2025) with high‐fiber or fruit‐based components that disrupt starch gelatinization.

**TABLE 4:** Pasting properties of composite flour made from cornstarch and *Mobola* plum flour at different ratios.

Sample	Peak viscosity (RVU)	Trough (RVU)	Breakdown (RVU)	Final viscosity (RVU)	Setback (RVU)	Pasting temperature (°C)
Control	3820.01 ± 3.4^f^	2650.01 ± 1.3^f^	1170.00 ± 1.2^f^	4120.04 ± 2.5^f^	1470.03 ± 1.3^f^	71.21 ± 0.4ᵃ
CPF1	3450.06 ± 3.5^e^	2460.03 ± 2.4^e^	990.03 ± 1.2^e^	3810.11 ± 1.7^e^	1350.07 ± 1.8^e^	72.62 ± 0.5ᵇ
CPF2	3010.11 ± 3.4^d^	2250.04 ± 2.3^d^	760.07 ± 1.1^d^	3490.12 ± 1.4^d^	1240.08 ± 1.5^d^	74.11 ± 0.6ᶜ
CPF3	2540.12 ± 3.4^c^	1980.05 ± 2.3^c^	560.07 ± 0.7^c^	3120.10 ± 2.3^c^	1140.05 ± 1.2^c^	75.81 ± 0.5ᵈ
CPF4	2080.14 ± 3.9^b^	1720.07 ± 1.9^b^	360.07 ± 0.9^b^	2760.16 ± 1.5^b^	1040.09 ± 1.2^b^	77.62 ± 0.7ᵉ
CPF5	1650.09 ± 2.5^a^	1420.06 ± 2.6^a^	230.03 ± 0.7^a^	2410.12 ± 1.3^a^	990.06 ± 0.8^a^	79.31 ± 0.6ᶠ

*Note:* The values are mean ± SD of triplicate determinations. Values followed by the same superscript(s) within the column differ significantly (*p* ≤ 0.05). CS (Control), CPF1 (90:10), CPF2 (80:20), CPF3 (70:30), CPF4 (60:40) and CPF5 (50:50). Cornstarch: *Mobola* Plum flour.

A similar decline in trough viscosity was noted, demonstrating stability loss in the paste during the high‐temperature holding period. The lesser trough viscosity in the composite samples suggests that with increasing levels of *Mobola* plum flour, the starch matrix became more susceptible to shear and thermal stresses (Ragoubi et al. 2022). Thus, the breakdown viscosity, derived from the difference between the peak and trough viscosities and reflecting the paste's fragility, dropped significantly from 1170.00 RVU in CS to 230.03 RVU in CPF5. The lower breakdown viscosities of the composite flours indicate that paste stability was improved, likely due to the structural non‐starch polysaccharide reinforcement that mitigated excessive granule fracture (Sharafi Zamir et al. 2025).

The ability of a paste to form a viscous gel upon cooling is measured as the final viscosity. Final viscosity consistently reduced as *Mobola* plum flour inclusion was increased. This reduced final viscosity indicates a reduced ability to reassociate starch molecules (Scott and Awika 2023), particularly high‐amylose starches, which are known to form stronger gels. Setback viscosity, which measures the tendency of starch to retrograde, also reduced from 1470.03 RVU in CS to 990.06 RVU in CPF5. Lower setback values in composite flours indicate reduced retrogradation (Park et al. 2025), which may lead to improved textural stability and reduced staling in the end products.

For viscosity parameters, a reduction of 71.21°C in CS to 79.31°C in CPF5 is a noteworthy increase in the pasting temperature range. An increase in *Mobola* plum flour substitution corresponds to an increased heating range for sample pasting. This is likely to hydrate the flour and starch components prior to gelatinization (Park et al. 2025). This also suggests the presence of starch‐polyphenol and/or starch‐fiber interactions. This will reduce swelling in the granules.

These findings show that incorporating *Mobola* plum flour significantly alters the pasting behavior of cornstarch. The combination of lower peak and final viscosities along with setback viscosities and higher pasting temperatures indicates that these composite flours might be suitable for food products that require lower final viscosities and improved stability to thermal processing and retrogradation, such as baby foods, bakery products, or extruded snacks. Nevertheless, higher levels of substitution might be detrimental to the formulation of products that require high thickening capacity.

### 3.5 Total Phenolic Content and Antioxidant Properties of Composite Flour Made From Cornstarch‐*Mobola* Plum (*Parinari curatellifolia*) Flour Blends

Total phenolic content (TPC) and antioxidant activity of composite flours derived from blends of cornstarch–*Mobola* plum (*Parinari curatellifolia*) flour are shown in Table 5. The incorporation of *Mobola* plum flour into cornstarch improves the phenolic content and antioxidant activity of composite flours (*p* < 0.05) compared with the control (100% cornstarch flour). The total phenolic content in the control sample was 33.75 mg GAE/g, and this increased to 91.09 mg GAE/g in CPF5. The increase is attributed to the high phenolic composition of *Mobola* plum flour. Moreover, it can be concluded that phenolic compounds, of which *Mobola* plum flour contains high levels, were transferred to the composite matrix. The contribution of phenolic compounds to antioxidant activity is due to their ability to donate hydrogen atoms (or electrons) and their ability to complex (chelate) pro‐oxidant metal ions (El‐Lateef et al. 2023).

**TABLE 5:** Total phenolic content and antioxidant properties of composite flour made from cornstarch‐*Mobola* plum flour blends.

SAMPLE	TPC (mg GAE/g)	ABTS (μmol TE/g)	FRAP (GAE/g)	DPPH (%)
Control	33.75 ± 0.9^a^	45.36 ± 0.9^a^	0.05 ± 0.0^a^	43.64 ± 0.8^a^
CPF1	42.43 ± 0.7^b^	64.47 ± 0.6^bc^	1.12 ± 0.1^b^	53.79 ± 0.3^b^
CPF2	52.66 ± 0.5^c^	64.49 ± 0.6^b^	1.49 ± 0.1^c^	66.57 ± 0.5^c^
CPF3	57.77 ± 0.3^d^	71.89 ± 0.9^cd^	1.45 ± 0.1^c^	71.44 ± 0.5^d^
CPF4	79.73 ± 0.6^e^	75.57 ± 0.8^d^	1.51 ± 0.1^c^	78.30 ± 0.7^e^
CPF5	91.09 ± 0.6^f^	74.92 ± 0.7^d^	1.90 ± 0.2^d^	84.82 ± 0.9^f^

*Note:* The values are mean ± SD of triplicate determinations. Values followed by the same superscript(s) within the column differ significantly (*p* ≤ 0.05). CPF1 (90:10), CPF2 (80:20), CPF3 (70:30), CPF4 (60:40) and CPF5 (50:50) Cornstarch (Control): *Mobola* Plum. TPC = total phenolic content. Values are mean ± standard deviation of triplicate determinations. Values followed by the same superscript(s) within the same column are not significantly different at *p* < 0.05.

A similar trend was observed in antioxidant activity, measured using the ABTS radical scavenging method. ABTS values in the control sample were at 45.36 μmol TE/g, while the samples that contained the least amount of *Mobola* plum flour (CPF1) had an increase in ABTS values (64.47 μmol TE/g), followed by CPF2 (64.49 μmol TE/g) and CPF3 (71.89 μmol TE/g). The sample containing the highest amount of *Mobola* plum flour (CPF4) measured at 75.57 μmol TE/g, and the other sample with high *Mobola* plum flour (CPF5) was measured at 74.92 μmol TE/g. The lack of a further increase in ABTS scavenging activity at the highest substitution level may be due to saturation, a lower reactivity of insoluble‐bound phenolic compounds to the ABTS radical, or the lack of accessibility of the phenolic compounds due to interaction with macromolecules that may be present in the sample (Shahidi and Hossain 2023). Additionally, the Ferric Reducing Antioxidant Power (FRAP) method showed significant increases with higher inclusions of *Mobola* plum flour. The control sample had an antioxidant capacity of 0.05 GAE/g, which was negligibly small, while samples containing *Mobola* plum flour had FRAP values ranging from 1.12 to 1.90 GAE/g, with CPF5 containing the highest value. There was a slight decrease in FRAP values between CPF2 and CPF3, but not significantly different (*p* < 0.05). The slight decrease may be attributed to insufficient matrix interaction during mixing. This trend demonstrated an increased electron‐donating capacity of the composite flours (Cheng et al. 2025) and highlighted the additional contribution of phenolic and other bioactive compounds in *Mobola* plums, which enhanced antioxidant activity.

Apart from the control, which displayed a lower inhibition value of 43.64%, the DPPH inhibition values increased progressively to 84.82% in CPF5. The increase in DPPH free radical scavenging activity also means that *Mobola* plum flour improved the free radical scavenging efficacy of the blends. The relationship between DPPH and TPC values confirms that the phenolic compounds were responsible for the antioxidant activity (Shahidi and Hossain 2023).

Results indicated a strong positive correlation between the inclusion of *Mobola* plum flour and the total phenolic content and antioxidant capacity of the composite flours. The composite flours also showed improved radical‐scavenging and reducing abilities, as evidenced by three antioxidant assays (ABTS, FRAP, and DPPH). These results suggest that *Mobola* plum flour can be successfully used in the formulation of starch‐based functional foods to help reduce oxidative stress.

Table 6 shows the cornstarch‐*Mobola* plum (*Parinari curatellifolia*) composite flour blends' color characteristics in the CIE L*a*b* color space and calculated variables (Δ*E*, chroma, hue angle, whiteness index, and yellowness index). There is a steady decrease in lightness (L*) from 94.96 in the control to 87.58 in CPF5, showing a significant darkening of the flour blends as more *Mobola* plum flour is added. Flours made with fruits as an ingredient tend to be darker, owing to their higher phenolic content, which contributes to greater light absorption (Islam and Hasan 2024; Ali et al. 2025). Composite flours containing plant‐based components such as fruit pulp, seed flour, and peel have also been reported to exhibit reduced L* values (Resende et al. 2020; Islam and Hasan 2024).

**TABLE 6:** Color analysis of composite flour made from Cornstarch‐*Mobola* plum flour blends.

Sample	Control	CPF1	CPF2	CPF3	CPF4	CPF5
L*	94.96 ± 0.9^a^	92.51 ± 0.7^b^	91.43 ± 0.8^c^	90.46 ± 0.9	88.84 ± 0.6^e^	87.58 ± 0.8^f^
a*	0.17 ± 0.0^a^	0.40 ± 0.1^b^	0.65 ± 0.0^c^	0.88 ± 0.0^d^	1.01 ± 0.1^e^	1.18 ± 0.2^f^
b*	4.23 ± 0.5^a^	7.08 ± 0.3^b^	10.02 ± 0.3	13.15 ± 0.2^d^	17.78 ± 0.2^e^	21.01 ± 0.02^f^
∆*E*	95.00 ± 0.7^a^	92.77 ± 0.9^b^	91.98 ± 0.7^c^	91.42 ± 0.9^d^	90.61 ± 0.7^e^	90.09 ± 0.8^f^
*C*	4.23 ± 0.6^a^	7.09 ± 0.3^b^	10.05 ± 0.4^c^	13.17 ± 0.3^d^	17.80 ± 0.2^e^	21.10 ± 0.3^f^
*H*°	87.7 ± 0.4^a^	86.74 ± 0.6^b^	86.25 ± 0.8^b^	86.25 ± 0.8^c^	86.74 ± 0.7^c^	86.78 ± 0.6^c^
WI	95.01 ± 0.6^a^	89.69 ± 0.7^b^	86.79 ± 0.7^c^	83.73 ± 0.9^d^	76.18 ± 0.7^e^	75.56 ± 0.3^f^
YI	6.36 ± 0.5^a^	10.93 ± 0.5^b^	15.66 ± 0.5^c^	20.75 ± 0.8^d^	28.58 ± 0.4^e^	34.28 ± 0.7^f^

*Note:* The values are mean ± SD of triplicate determinations. Values followed by the same superscript(s) within the rows differ significantly (*p* ≤ 0.05). CPF1 (90:10), CPF2 (80:20), CPF3 (70:30), CPF4 (60:40) and CPF5 (50:50). Cornstarch (Control): *Mobola* Plum and cornstarch were used as controls. *L* (lightness) axis—0 is black, 100 is white, *a* (red–green) axis—positive values are red; negative values are green and 0 is neutral, *b* (yellow–blue) axis—positive values are yellow; negative values are blue and 0 is neutral, Δ*E* is total color change, *C* is chroma (color intensity), *H*° (Hue angle color saturation), WI is whiteness index and YI is yellowness index.

The a* values changed from 0.17 (control) to 1.18 (CPF5), indicating a shift towards red chromaticity across all samples as *Mobola* plum flour was added. Even though the a* values are statistically insignificant, the low values indicate that there are naturally occurring reddish‐brown pigments, which could be from the polyphenols and flavonoids in *Mobola* plum pulp (Fakudze et al. 2023). In earlier studies, similar increases in a* values have been observed due to higher levels of bioactive ingredients in composite and functional flours (Alija et al. 2025). Aside from that, the b* values also increased across all samples, from 4.23 in the control to 21.01 in CPF5, making the flour blends appear more yellow. This is the same case as the increased yellow index (YI) from 6.36 to 34.28. The great increases of b* and YI values are due to the yellow and orange pigments in the *Mobola* plum fruit (Xu et al. 2025). Increased yellowness was observed in all cereal and fruit composite flours, and across all studies, the authors determined which flours increased provitamin A concentrations despite a decrease in visual whiteness (Abayomi et al. 2023).

Color difference (Δ*E*) values decreased gradually from 95.00 in the control sample to 90.09 in CPF5. Despite the reference white standard yielding high Δ*E* values across all samples, statistically significant differences indicate that the blends exhibited perceptible color differences. Δ*E* values exceeding 3 are, according to color perception thresholds, considered distinguishable, indicating that *Mobola* plum flour incorporation will result in color changes that will be obvious in flour‐based products (Malengue et al. 2024). Chroma (C), describing intensity or saturation of a color, increased significantly with increased substitution levels from 4.23 in the control to 21.10 in CPF5. This increase indicates a greater color saturation in composite flours with higher levels of *Mobola* plum flour. Greater chroma is commonly linked to greater pigment content and less dilution from white starch matrices (Su et al. 2024).

The hue angle (*H*°) values ranged from 86° to 88°, suggesting that the dominant color remained constant within the yellow color region. This indicates that the addition of *Mobola* plum flour did not affect the dominant hue of the flour blends, despite variations in color brightness and intensity. With increased substitution of plum flour in flour blends, the whiteness index (WI) decreased significantly from 95.01 in the control to 75.56 in CPF5, indicating a loss of whiteness. Whiteness is important in flour intended for use in Bakery and Confectionery because it impacts the consumer acceptance of the product (Garvey et al. 2020). The decreased WI is consistent with reports in composite flour made from fruits or legumes (Weerarathna and Wansapala 2024; Akinwotu et al. 2025).

In general, the color changes seen in the cornstarch–*Mobola* plum composite flours show that the increased inclusion of *Mobola* plum flour changes the appearance of the flour by decreasing the lightness and whiteness of the flour and increasing the yellowness and color saturation. These changes might be limiting for whiteness‐demanding applications, but for food applications that require nutritional profile enhancement or for speciality food products, they might be beneficial, particularly when the acceptability or desirability of darker or yellowish colors is present.

## 4 Conclusion

This study shows that the addition of *Mobola* plum flour alters the pH and soluble solids of cornstarch‐based composite flours, with little effect on titratable acidity. These changes imply that *Mobola* plum flour could improve composite flours and, therefore, support its use as a value‐adding ingredient in the formulation of new food products. Additionally, incorporating *Mobola* plum flour decreases moisture and fat content while increasing ash, enabling the creation of composite flours that are low in fat and high in nutrients. From the changes, it is observed that CPF1‐CPF3 provides the best proximate composition, functional properties and pasting.

## Author Contributions

**Shonisani E. Ramashia:** conceptualization, supervision, writing – review and editing. **Winnie Ndou:** data curation, writing – original draft, formal analysis. **Oluwaseun P. Bamidele:** conceptualization, validation, writing – review and editing, formal analysis.

## Disclosure

Author approval and responsibility statement: All authors have read and approved the final version of the manuscript. The corresponding author had full access to all data in this study and takes full responsibility for the integrity and accuracy of the data analysis.

## Conflicts of Interest

The authors declare no conflicts of interest.

## Data Availability

The data supporting the findings of this study are available from the corresponding author upon reasonable request.
